# Be Neighborly

**Published:** 1871-11

**Authors:** 


### BE NEIGHBORLY.

Be sociable; strive to be on visiting terms with the families who live nearest you, and to gain an influence over them; this can be the more easily done if you are rich; in this case, wealth is a talent; it is an instrumentality for good which every man and woman is bound to use to advantage; for a talent unused, a talent unemployed, brings the disapprobation of the Infinite One. As long as the world stands, the masses will look up to wealth; they will do things to please the rich; will be at pains to get their commendation, their recognition. Suppose the rich men and women of any church were to cultivate a certain degree of sociableness and friendliness, of neighborly courtesy toward all living within a mile or two of the meeting house, it is perfectly certain that that house would be more encouragingly filled on Sundays than otherwise.

But then a good, a physical good, would come to the good doer. The visit to the poor man's dwelling involves exercise in the open air; by talking with his family, we get new views, new ideas; have different phases of life presented to us, and new feelings and new emotions come across the mind to wake up the powers within us to healthful activities.

In the same connection it is advantageous for all, sick or well, to

MIX AMONG STRANGERS.

It breaks up the wearing monotony of home life; breaks up that stagnation of thought and feeling and emotion which attends a life of sameness and inactivity.

